# A Pathophysiologic Approach to Biomarkers in Acute Respiratory Distress Syndrome

**DOI:** 10.1155/2016/3501373

**Published:** 2016-02-11

**Authors:** Raiko Blondonnet, Jean-Michel Constantin, Vincent Sapin, Matthieu Jabaudon

**Affiliations:** ^1^CHU Clermont-Ferrand, Intensive Care Unit, Department of Perioperative Medicine, Estaing University Hospital, 63000 Clermont-Ferrand, France; ^2^Clermont Université, Université d'Auvergne, EA 7281, R2D2, 63000 Clermont-Ferrand, France; ^3^Department of Medical Biochemistry and Molecular Biology, CHU Clermont-Ferrand, 63000 Clermont-Ferrand, France

## Abstract

Acute respiratory distress syndrome (ARDS) is an acute-onset hypoxic condition with radiographic bilateral lung infiltration. It is characterized by an acute exudative phase combining diffuse alveolar damage and lung edema followed by a later fibroproliferative phase. Despite an improved understanding of ARDS pathobiology, our ability to predict the development of ARDS and risk-stratify patients with the disease remains limited. Biomarkers may help to identify patients at the highest risk of developing ARDS, assess response to therapy, predict outcome, and optimize enrollment in clinical trials. After a short description of ARDS pathobiology, here, we review the scientific evidence that supports the value of various ARDS biomarkers with regard to their major biological roles in ARDS-associated lung injury and/or repair. Ongoing research aims at identifying and characterizing novel biomarkers, in order to highlight relevant mechanistic explorations of lung injury and repair, and to ultimately develop innovative therapeutic approaches for ARDS patients. This review will focus on the pathophysiologic, diagnostic, and therapeutic implications of biomarkers in ARDS and on their utility to ultimately improve patient care.

## 1. Introduction

The acute respiratory distress syndrome (ARDS) is a heterogeneous syndrome defined by the association of bilateral radiographic pulmonary opacities, arterial hypoxemia (partial pressure of arterial oxygen (PaO_2_) to fraction of inspired oxygen (FiO_2_) ratio <300 with a positive end-expiratory pressure of 5 cm H_2_O or more), and exclusion of cardiac failure as a primary cause [1]. It is characterized by diffuse alveolar epithelial and lung endothelial injury leading to increased permeability pulmonary edema and alveolar filling [2]. By definition, ARDS occurs within one week of a known clinical insult or new or worsening respiratory symptoms, as a consequence of various risk factors including either direct (e.g., bacterial or viral pneumonia, gastric aspiration, lung contusion, toxic inhalation, and near drowning) or indirect (e.g., sepsis, pancreatitis, severe trauma, massive blood transfusion, and burn) lung injury [1]. Despite improvements in intensive care during the last fifteen years, ARDS is still a frequent (60/100000 inhabitants/year), morbid, and life-threatening condition, with a mortality rate around 30% [3–5]. In addition, there has been recent recognition of the clinical and biological heterogeneity within ARDS [6–8], thus reflecting our incomplete understanding of the biology of ARDS and hampering the successful clinical translation of new diagnostic, preventive, and therapeutic strategies [9]. Some investigators have further proposed subdividing ARDS, for example, on the basis of clinical risk factors [10], by direct versus indirect lung injury [7], or by focal versus nonfocal lung morphology as assessed by CT-scan [11, 12]. Characterizing ARDS phenotypes may help to better understand genetic, genomic, and protein risk factors for ARDS, predict the syndrome, identify mechanism-defined subgroups of ARDS, and/or to better target therapy [10, 13]. The subtype (or phenotype) of a condition is ideally defined by a distinct functional/pathobiological mechanism, named endotype, that may explain, at least in part, response to treatment [13].

## 2. Pathogenesis of ARDS

The pathogenesis of ARDS is characterized by two phases that may sometimes overlap temporally and spatially [2]: exudative and proliferative [14] phases. An alveolar-capillary barrier dysfunction resulting in altered permeability of epithelial and endothelial alveolar cells characterizes the early exudative phase. Due to loss of cellular integrity, alveoli are filled with proteinaceous edema fluid that results in impaired gas exchange. Initially, there is an early exudative phase associated with diffuse alveolar damage, microvascular injury with subsequent pulmonary edema, alveolar type 1 (AT1) epithelial cell necrosis, and influx of inflammatory cells which then release active mediators [2]. During this early phase, alveolar inflammation is mainly mediated by polymorphonuclear neutrophils (PMN) [2], but recent findings also support a key role for monocytes and macrophages [15, 16]. Other proinflammatory mechanisms are also involved, as the significant release of proinflammatory cytokines by lungs cells, inflammatory cells, and fibroblasts.

The association of persistent injury and failure to repair lung damage in a timely manner mainly contributes to the pathological fibroproliferative response during which there are proliferation of fibroblasts, hyperplasia of AT2 cells, and lung repair. The repair of the injured alveolar epithelium remains incompletely understood; it involves hyperplasia of AT2 (and maybe AT1) cells, migration along the basement membrane by AT2 cells to form a new epithelial barrier, and complex interactions with ECM and other cells including alveolar macrophages. In the absence of recovery, processes leading to fibrosing alveolitis may occur during a fibrotic phase, resulting in some cases in marked changes in lung structure and function [17].

## 3. Biomarkers of ARDS: A Pathophysiologic Approach

The discovery and validation of biomarkers of myocardial injury and ventricular overload such as troponin and brain-natriuretic peptide (BNP) have transformed the diagnosis, management, and design of clinical trials in conditions such as myocardial infarction and congestive heart failure [18, 19]. In a similar way, identification of plasma biomarkers that may facilitate diagnosis of ARDS could, at least in theory, improve clinical care, enhance our understanding of pathophysiology, and be used to enroll more homogeneous groups of patients in clinical trials of new therapies, increasing the likelihood of detecting a treatment effect [20]. Pathophysiologic changes can probably be used as a framework to better understand various biomarkers that have been studied in ARDS, including the cellular injury pathways that are central to lung injury: endothelial injury, epithelial injury, proinflammatory injury, coagulation, fibrosis, and apoptosis [21].

Among multiple potential applications, biomarkers may be used to better identify patients with risk factors of ARDS who are most likely to develop the syndrome. Subsequently, they may also be useful to improve risk stratification once ARDS criteria are present. Biomarkers may also play a pivotal role in the design of future clinical trials through the identification of patients at high risk of poor outcome, thus decreasing the required sample size needed to show a therapeutic benefit [92]. More recently, biomarkers have also been proven useful to evaluate the response to therapy [13, 93]. Finally, the study of biomarkers in ARDS plays a fundamental role in understanding the mechanisms and pathophysiology underlying lung injury, thus serving as a solid basis to develop future therapeutic strategies [9].

**Figure 1:**
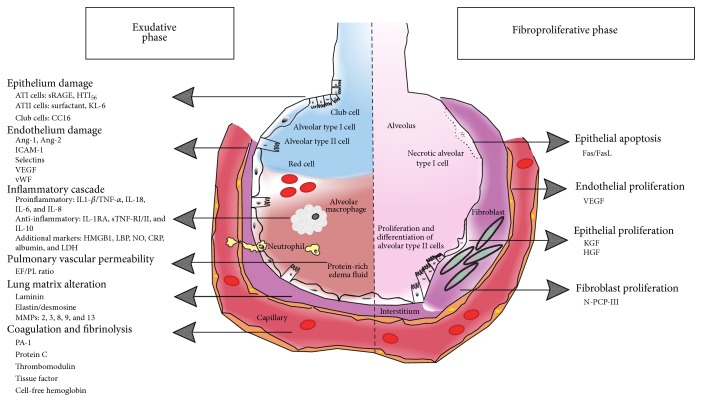
Biomarkers of acute respiratory distress syndrome organized by pathways and phases of lung injury (left: early exudative phase; right: fibroproliferative phase).

We will now review the biomarkers that have been investigated in ARDS, with a focus on biomarkers in groups that reflect their primary function (Figure 1 and Table 1).

### 3.1. Exudative Phase of ARDS

ARDS is characterized by an initial exudative phase with diffuse alveolar damage associated with the formation of lung inflammatory edema. Alveolar injury is predominant during this phase, and various proteins that are specific to lung injury are therefore released in both the blood and the alveolar compartment, thus serving as markers of the disease or of its resolution.

#### 3.1.1. Lung Injury

*(1) Alveolar Epithelium*. The alveolar epithelial lining is composed of AT1 and AT2 epithelial cells and plays a critical role in barrier function, regulating surfactant production, and in vectorial transport of alveolar fluid. During the acute phase of ARDS, alveolar epithelial cells undergo neutrophil-mediated damage and effects of proinflammatory cytokines or of hypoxic injury, thus accounting for the clinical syndrome [2]. Lung-secreted proteins may be found in both the bronchoalveolar (BAL) fluid and the systemic blood because they can move passively across the epithelial barrier into serum where they may serve as peripheral indicators of epithelial damage. Several markers of lung epithelial damage have been studied as markers of ARDS, as supported by the fact that they should be more specific to lung injury than other markers, for example, inflammatory cytokines [94].

*(a) Alveolar Type 1 Cells*. They are of two types.

*RAGE*. AT1 epithelial cells cover 90–95% of the alveolar surface and contribute to both alveolar fluid clearance (AFC) and barrier integrity. The receptor for advanced glycation end-products (RAGE) is a transmembrane pattern-recognition receptor of the immunoglobulin superfamily that is constitutively expressed at low levels in all cells but abundantly in the lung. RAGE is primarily located on the basal surface of AT1 cells [22, 23]. Activation of RAGE modulates cell signaling, culminating in a sustained inflammatory response through various intracellular signaling pathways such as cytokines, reactive oxygen species (ROS), or proteases and leading to proinflammatory activation of nuclear transcription factor NF-*κ*B [95, 96]. RAGE is implicated in ARDS as an important pathway to innate immunity and alveolar inflammation [97] and when the soluble form (sRAGE, for soluble RAGE) is assayed in plasma or pulmonary edema fluid, as a marker of alveolar injury [98–101]. Full-length RAGE is a transmembrane receptor, but it can also be found as soluble isoforms, generally referred to as soluble RAGE (sRAGE, comprising the extracellular domain of RAGE and produced through the cleavage of full-length RAGE by matrix metalloproteinases) [102, 103] and endogenous secretory RAGE (esRAGE, produced after alternative splicing) [104]. Full-length RAGE and its isoforms are abundantly and constitutively expressed in the lungs in normal conditions [103, 105–107], and sRAGE is now considered as a promising novel marker of AT1 cell injury and a key mediator of alveolar inflammation [22, 95, 108]. It is shown that sRAGE expression appears enhanced during the early stage of ARDS. Our team, with others, has recently reported in both ARDS patients and a mouse model of ARDS that the extent of sRAGE elevation in plasma and alveolar fluid correlates with markers of severity assessed by PaO_2_/FiO_2_, lung injury, and alveolar fluid clearance (AFC) [98–101, 109]. A role for RAGE pathway in the regulation of AFC has been recently described for the first time [110] and is under active investigation by our team and others [101, 111]. Interestingly, plasma and BAL sRAGE levels are elevated during ARDS, independently of any associated severe sepsis [100]. In addition, plasma levels of sRAGE are correlated with diffuse damage as assessed by lung CT-scan and are correlated with the extent of alveolar damage [100, 112], suggesting that sRAGE may serve as a useful biomarker of AT1 cell injury and lung damage during ARDS. Plasma levels of sRAGE are also associated with 28-day and 90-day mortality in patients with ARDS [99, 106, 112].

Calfee et al. recently compared biomarker levels in patients with direct versus indirect ARDS enrolled in a single center study of 100 patients and in a secondary analysis of 853 ARDS patients drawn from a multicenter randomized controlled trial [7]: levels of biomarkers of lung epithelial injury (sRAGE, surfactant protein-D) were significantly higher in direct ARDS compared to indirect ARDS.

A recent observational study also supports an ARDS phenotype based on levels of RAGE ligands and soluble forms, as elevated sRAGE, high mobility group box-1 protein (HMGB1), and S100A12, with decreased esRAGE and advanced glycation end-products (AGEs), were found to distinguish patients with ARDS from those without [109]. Although these recent findings warrant further validation in multicenter studies, monitoring sRAGE levels may be useful in assessing the response to strategies in ventilator settings including alveolar recruitment maneuvers in patients with ARDS [113], or in patients without lung injury at risk of postoperative respiratory complications after major surgery [24]. The predictive value of 4 single-nucleotide polymorphisms in RAGE gene (AGER) in the development of ARDS in at-risk patients is currently under study by our team [114]. 

*HTI*_*56*_. Human type I cell-specific membrane protein (HTI_56_) is a 56-kDa glycosylated lung protein specific to the apical membrane of human AT1 cells. HTI_56_ has biochemical characteristics of an integral membrane protein [25]. Although the precise functions of HTI_56_ remain unknown, HTI_56_ is an analog to RTI_40_, a 40–42 kDa integral membrane protein specific to the apical membrane of rat AT1 cells [26]. Patients with ARDS had higher levels of HTI_56_ in both lung edema fluid and plasma as compared to patients with hydrostatic lung edema [94], but no study assessing the association between HTI_56_ levels and other endpoints in patients with ARDS (e.g., prognosis) has been published to date. 

*(b) Alveolar Type 2 Cells*. AT2 cells have important homeostatic functions in the lung, including AFC and production of alveolar surfactant (involved in lung compliance, keeping the alveolus open). AT2 cells are also known as key mediators of the epithelial repair process [115]. 

*Surfactant Proteins*. Surfactant has a vital role in maintaining the integrity of the alveolar-capillary interface. Its essential function is to decrease surface tension into the alveoli, thus stabilizing lung volume at low transpulmonary pressures. Surfactant is composed of approximately 80% phospholipids, 8% other lipids (cholesterol, triacylglycerol, and free fatty acids), and 12% proteins. Four surfactant-associated proteins (SP), designated SP-A, SP-B, SP-C, and SP-D, represent approximately half of proteins composing surfactant. SP-A and SP-D easily dissociate from lipids and are hydrosoluble. They belong to the lung innate immune system, thereby enhancing phagocytosis of bacteria and virus. They also exert regulatory effects on AT2 cells. SP-B and SP-C are small, extremely hydrophobic proteins that are important in the formation of the surfactant monolayer in the terminal airspaces and in the reduction of surface tension, thus preventing end-expiratory alveolar collapse [116].

Early observations in ARDS revealed a loss in surface tension suggesting a functional loss of the surfactant proteins [117]. In a first case report of three patients, the ratio of plasma SP-B/SP-A was inversely associated with both blood oxygenation and static respiratory system compliance, suggesting that SP-B breaches the alveolocapillary barrier more readily than SP-A and may therefore provide a more sensitive marker of lung injury [118]. Plasma levels of SP-A and SP-B are increased in patients with ARDS [70] and in at-risk patients [119, 120], whereas lower SP-A and SP-B levels were found in the BAL fluid of patients at risk for ARDS prior to the onset of the clinical syndrome. SP-A and SP-B levels remained low for as long as 14 days in patients with sustained ARDS. Interestingly, this decrease in BAL SP-A and SP-B does not result simply from dilution of alveolar fluids by plasma entering the alveolar spaces, as SP-D levels remained stable in parallel [119]. In a cohort of 38 patients, reduced pulmonary edema fluid SP-D and elevated plasma SP-A at the onset of ARDS were associated with poor prognosis [27]. Nevertheless, in a study of 259 patients from the ARDSNet trial of low versus high end-expiratory pressure in ARDS (ALVEOLI) as well as in 75 patients enrolled in a randomized trial of activated protein C for ARDS, plasma SP-D was not associated with 28-day mortality or ventilator-free days [28]. 

*KL-6*. Krebs von den Lungen-6 (KL-6) is a human MUC1 mucin that belongs to the high-molecular-weight glycoprotein family. After the cleavage of S-S bond, KL-6 can spread into the pulmonary epithelial lining fluid. In the normal lung, this glycoprotein can be predominantly found in AT2 cells, and its expression is enhanced during AT2 proliferation, regeneration, or injury, thus representing an attractive biomarker in ARDS. Plasma KL-6 is elevated in ARDS patients and correlates with lung injury and mortality [29, 121–123]. Plasma levels of SP-D and KL-6 increase over time in patients with ARDS and may represent biological markers of ventilator-associated lung injury because their increase is attenuated by lung-protective ventilation [30]. 

*(c) CC16*. Clara cell protein (CC16) is a 15.8 kDa homodimeric protein that is abundantly secreted in airways by the nonciliated bronchiolar Clara cells. Clara cells are devoted to the protection of the respiratory tract against toxic inhaled agents, the repair of damaged epithelium, xenobiotics detoxification, and the secretion of proteins with important biological activities. CC16 is highly expressed in the epithelial lining fluid, with antioxidant/inflammatory roles, notably by modulating the production and/or activity of phospholipase-A2, interferon-*γ*, and tumor necrosis factor-*α* [31]. Available studies found contradictory, inconclusive findings during ARDS. Although higher levels of CC16 are associated with lung injury and inflammation in some experimental and clinical studies, patients with ARDS had lower plasma and pulmonary edema fluid levels of CC16 than patients with acute cardiogenic pulmonary edema, and no correlation was found between CC16 and prognosis. So many conflicting findings do not currently support the association of CC16 with the diagnosis or prognosis of ARDS.

*(2) Vascular Endothelium*. Vascular endothelial injury is characterized by the disruption of cell components leading to increased microvascular permeability and alveolar edema. Endothelial injury is mainly driven by the activation of inflammation and coagulation cascades. The activation of endothelial cells by circulating mediators leads to increased expression of cell surface molecules that are important mediators of leukocyte adhesion and contribute to leukocyte accumulation and transmigration [124]. Activated lymphocytes can also release mediators in microvessels that increase vascular permeability. Along with these leukocyte signals, inflammatory mediators such as tumor necrosis factor (TNF), thrombin, and vascular endothelial growth factor (VEGF) disrupt endothelial-cadherin bonds and contribute to the vascular leak underlying edema formation in ARDS. Platelets also contribute to endothelial injury through the release of cytokines and through fibrin clotting [92]. 

*(a) Angiopoietin*. Angiogenic agents, along with VEGF and angiopoietin-1 (Ang-1), play key roles in vascular development. VEGF stimulates the generation of new, immature, and leaky blood vessels whereas Ang-1 enhances angiogenesis, inducing vascular maturation, and decreases vascular permeability [33]. The most encouraging data result from recent studies of angiopoietin-2 (Ang-2), an endothelial protein that has been studied extensively during sepsis [125]. Ang-2 has an important role as it increases endothelial junction instability, enhances vascular leak, naturally antagonizes Ang-1, and, in the absence of other angiogenic stimuli, induces vascular regression and endothelial cell apoptosis. Both Ang-1 and Ang-2 are ligands for the tyrosine kinase receptor Tie-2 [126], and a link between Ang-2 and inflammation has been reported [127]. Therefore, such vascular growth factors have been proposed as biomarkers for ARDS [128]. First, two single-nucleotide polymorphisms within the Ang-2 gene (rs1868554 and rs2442598) were associated with the risk of developing ARDS in trauma patients [129]. In addition, Agrawal et al. found in a prospective study of 230 patients admitted to the intensive care unit (ICU) without ARDS that higher levels of Ang-2 were significantly associated with increased development of ARDS [130]. In surgical ICU patients, levels of Ang-2 were higher in patients with ARDS than in those without the syndrome [131]. A higher Ang-2/Ang-1 ratio was also an independent predictor of mortality in ARDS patients [131, 132], and patients with infection-related ARDS whose Ang-2 levels increased between day 0 and day 3 doubled their odds of death, suggesting that Ang-2 kinetics may be particularly valuable by reflecting evolving lung injury [34]. Finally, in a large study of 931 patients enrolled in the ARDSNet fluid and catheter treatment trial, baseline plasma levels of Ang-2 were associated with 90-day mortality in patients with noninfectious ARDS, whereas this association was not found in patients with infection as their primary ARDS risk factor. Based on a secondary analysis of two large studies, Calfee et al. further demonstrated that indirect lung injury is characterized by a molecular phenotype consistent with more severe lung endothelial injury, as assessed by plasma Ang-2, and less severe epithelial injury [7]. 

*(b) ICAM-1*. The soluble intercellular adhesion molecule-1 (sICAM-1) is an inducible glycoprotein expressed on the surface of vascular endothelial cells and other cells (e.g., hematopoietic cells, AT1 cells) [35]. Under physiologic conditions, sICAM-1 is not constitutively expressed or is expressed at low levels in most tissues. During inflammation, and in response to stimuli such as interferon-*γ* (IFN-*γ*) or interleukin-1 (IL-1), levels of sICAM-1 are upregulated [133]. Elevated levels have also been found in both plasma and lung edema fluid from patients with ARDS, as compared to patients with hydrostatic lung edema [134]. In a multicenter study, the increase of sICAM-1 from baseline to day 3 was associated with poor clinical outcome [135], and in a prospective cohort of 65 patients with ARDS, baseline plasma levels of sICAM-1 were also associated with mortality [136]. In pediatric patients with ARDS, early elevated plasma levels of sICAM-1 were associated with increased risks of death and of prolonged mechanical ventilation [137]. In trauma patients, higher plasma levels of sICAM-1 at baseline were correlated with future development of multiple organ dysfunction syndrome (MODS) but not with the development of ARDS [138–140]. 

*(c) Selectins*. Selectins are membrane-associated glycoproteins that mediate the adhesion of leukocytes and platelets to vascular surface. L-selectin is mainly expressed by leukocytes. P-selectin is rapidly redistributed from membranous secretory granules to the surface of activated platelets and endothelial cells [36]. E-selectin is expressed by cytokine-activated endothelial cells. It has been shown that plasma levels of such soluble adhesion molecules were markedly higher in nonsurvivors among critically ill patients and that they were negatively correlated with lung function (e.g., PaO_2_/FiO_2_ ratio) [141]. Other studies found that plasma P-selectin was elevated in patients with ARDS, especially in those who subsequently died, as compared with patients with other pulmonary diseases or sepsis but without ARDS [142]. Interestingly, patients with ARDS with chronic alcohol consumption had elevated levels of soluble E-selectin in both the plasma and epithelial fluid consistent with altered endothelial and alveolar-capillary function [143]. More recently, E-selectin was measured in the plasma levels from 50 individuals admitted to the emergency department and who were at-risk for developing ARDS, with higher E-selectin levels being associated with both ARDS development and 28-day mortality [144]. Circulating soluble E-selectin levels were elevated in pneumonia patients with ARDS, and plasma levels decreased along with the treatment of pneumonia [37]. 

*(d) VEGF*. One of the most extensively studied endothelial markers in ARDS is VEGF, albeit its value as a biomarker remains unclear. Vascular endothelial growth factors belong to the platelet-derived growth factor supergene family. They play central roles in the regulation of angiogenesis and lymphangiogenesis [38]. Alternative splicing of the VEGF gene (6p21.3) transcript leads to the generation of several splice variants, or isoforms, with various sizes [145]. VEGF-A is a 34–46 kDa glycoprotein acting as the major factor implicated in angiogenesis. It binds to two tyrosine kinase (TK) receptors, named VEGFR-1 (Flt-1) and VEGFR-2 (KDR/Flk-1), and regulates endothelial cell proliferation, migration, vascular permeability, secretion, and other endothelial functions [146]. The expression of VEGF in ARDS varies, depending on the degree of epithelial and endothelial damage. Many lung cells release VEGF, for example, AT2 cells, neutrophils, alveolar macrophages, and activated T cells. Thus, VEGF is potentially capable of having an effect on both alveolar epithelial and endothelial barriers. Interestingly, overexpression of VEGF induces pulmonary edema in animal models [147]. In a single center study, plasma levels of VEGF were increased in subjects with ARDS, compared to controls, and elevated plasma VEGF as measured on day 4 was associated with mortality in patients with ARDS [83]. Nevertheless, several studies suggest that plasma and alveolar VEGF may help to predict the development of ARDS and its recovery. Whereas plasma VEGF is increased in ARDS patients, VEGF levels were decreased in the BAL fluid from ARDS patients, as compared to controls [84, 148, 149]. In order to better understand such differences between plasma and alveolar expression of VEGF, Ware et al. conducted a study with the aim to determine whether changes in alveolar levels of VEGF were specific to ARDS or not [39]: the authors found that alveolar levels of VEGF were decreased in both patients with ARDS and those with hydrostatic edema. The mechanisms implicated in this alveolar decrease in VEGF during ARDS might not depend on the degree of lung injury but rather on the degree of alveolar flooding [39]. 

*(e) vWF*. Early studies of endothelial markers focused on von Willebrand Factor (vWF), a macromolecular antigen that is produced predominantly by endothelial cells, and to a lesser extent by platelets. In the setting of endothelial activation or injury, vWF is released from preformed stocks into the circulation [40, 41]. VWF has been investigated as a biological marker of endothelial injury in patients both at-risk for ARDS and with established ARDS [40, 150]. In a prospective study of 45 ICU patients with sepsis, patients with nonpulmonary sepsis had higher levels of plasma vWF, with good predictive and prognostic values for ARDS. Indeed, elevated plasma levels of vWF had a sensitivity of 87% and a specificity of 77% for the prediction of ARDS development in the setting of nonpulmonary sepsis [151]. However, subsequent studies in patients at-risk for ARDS did not confirm these findings [42, 152, 153]. In another study of 559 patients with ARDS enrolled in the National Heart, Lung, and Blood Institute ARDS Network trial of lower tidal volume, nonsurvivors had higher plasma levels of vWF, compared to survivors [40]. Higher vWF levels were significantly associated with fewer organ failure-free days, suggesting that the degree of endothelial activation and injury is strongly associated with outcomes in ARDS; nevertheless, ventilator settings had no impact on vWF levels in this study [40]. 

*(f) Lung Extracellular Matrix*. The extracellular matrix (ECM) forms the region of the lung situated between the alveolar epithelium and the vascular endothelium. ECM plays a mechanical role as it supports and maintains tissular structures. ECM also represents a complex and dynamic meshwork influencing many biological cell functions such as development, proliferation, and migration [154]. Collagens are the main component of ECM, along with glycoproteins and proteoglycans including hyaluronic acid. 

*Laminin*. Laminins (LM) are ECM proteins with high molecular weights that deposit in basal membranes. Laminins are involved in cell processes such as cellular adhesion, growth, and differentiation [43]. Laminin-5 (LM-5) plays an important role in cell migration and in the remodeling of epithelial tissue. LM-5 is activated through its cleavage by matrix metalloproteinases (MMPs), thus releasing a soluble LN *γ*2 NH2-terminal fragment (G2F) that does not deposit in the ECM and can therefore be detected in the peripheral blood. In a small single center study, laminin was measured in the plasma and lung edema fluid from 17 patients with ARDS, with higher levels found in patients with ARDS as compared to healthy volunteers [44]. Interestingly, nonsurvivors had higher plasma levels of laminin, as measured 5 days after ARDS onset, than survivors, and survivors had decreasing levels of the marker over time, suggesting that its secretion is suppressed during ARDS recovery. 

*Elastin/Desmosine*. Elastin is another critical protein of the ECM that gives the lung its elastic recoil ability. In adults, elastin, which is expressed by lungs and other tissues, is usually excreted in the urine. During lung epithelial and endothelial injury, elastin can be broken down by proteases such as neutrophil elastase [45]. Elastin breakdown results in smaller fragments containing desmosine and isodesmosine [155]. In a large study of 579 patients with ARDS, those ventilated with lower tidal volumes had lower urine desmosine levels, a finding that may reflect reduced extracellular matrix breakdown; however, no correlation with mortality was found in patients with ARDS [46]. 

*MMPs*. Matrix metalloproteinases (MMPs) are zinc-dependent endopeptidases that are able to degrade almost all extracellular matrix components. MMP-8, a member of the leukocyte-derived MMPs, contributes to the degradation, turnover, and remodeling of the extracellular matrix digesting type I collagen [47, 48]. MMPs are major actors in almost all phases of the inflammatory response, and their function is highly regulated. At the tissue level, most important inhibitors are the tissue inhibitors of metalloproteinases (TIMPs). In fulminant inflammation, the inhibitory capacity of TIMPs may be overwhelmed, leading to excessive tissue damage and adverse outcome [47]. Previous studies suggest that MMPs may have an important role in ARDS, although this role may be either harmful or beneficial [156, 157]. In a recent study, despite MMP-8 levels did not predict outcome in ARDS patients, higher levels of TIMP-1 were independently associated with increased 90-day mortality in a large group of critically ill, mechanically ventilated patients [47]. These findings are in contradiction to those from a study in pediatric ARDS patients in which higher MMP-8 and active MMP-9 levels, as measured 48 hours after disease onset, were associated with longer durations of mechanical ventilation and fewer ventilator-free days [158]. Elevated MMP-2, MMP-8, and MMP-9 in the BAL fluid from ARDS patients were associated with patterns of acute inflammation but with poor outcome [157]. Interestingly, MMP-3 and MMP-13 may be protective against lung injury by cleaving transmembrane receptor RAGE into sRAGE, thus regulating RAGE activation by its ligands [48, 49].

#### 3.1.2. Inflammatory Cascades

During ARDS, inflammatory responses can either be related to an ongoing primary infectious stimulus such as pneumonia or to systemic inflammation, such as in sepsis or in pancreatitis [2]. The inflammatory cascade involves inflammatory cells and the release of inflammatory mediators, as driven by a complex network of cytokines. A comparison between blood and alveolar cytokines suggests that most inflammatory mediators originate from the lung [17]. Alarmins, or damage-associated molecular patterns (DAMPs), are released by dead cells or local inflammatory cells (e.g., alveolar macrophages). They activate and recruit immune cells via binding to different receptors, such as TLR, IL-1 receptor (IL-1R), or RAGE, thereby initiating and perpetuating multiple proinflammatory pathways [21, 159].

Regulation of the inflammatory response is a complex process that requires interplay between several immune mediators [160]. Both pro- and anti-inflammatory biomarkers have been studied in ARDS. 

*(1) Proinflammatory Cytokines*

*(a) IL-1β and TNF-α*. IL-1*β* and TNF-*α* are the most biologically potent cytokines secreted by activated macrophages in the early phase of ARDS. They cause the release of a variety of proinflammatory chemokines such as monocyte chemotactic protein-1 (MCP-1), macrophage inflammatory protein-1*α* (MIP-1*α*), IL-6, and IL-8 with subsequent recruitment of inflammatory cells into the air spaces, alteration of the endothelial-epithelial barrier permeability, and impairment of fluid transport leading to alveolar edema [17]. TNF-*α* also promotes lung edema indirectly, through the production of reactive oxygen species (ROS) and a decreased expression of epithelial sodium (ENaC) and Na^+^-K^+^-ATPase channels [161]. Finally, TNF-*α*, a potent chemoattractant for fibroblasts, is a promoter of lung fibrosis in experimental studies [162, 163]. Interestingly, the ratio of BAL to serum levels of both TNF-*α* and IL-1*β* is typically high, suggesting that such cytokines may originate from the lung in the setting of ARDS [164]. Persistent elevation of plasma and BAL IL-1*β* is associated with worse outcome [165, 166]. Both TNF-*α* and IL-1*β* are elevated in the plasma and BAL fluid from patients at risk of and with ARDS [164, 167] and associated with mortality [166]. 

*(b) IL-18*. Inflammasomes are intracellular macromolecular complexes that serve as platforms for the activation of the proinflammatory enzyme caspase-1, which in turn cleaves pro-IL-1*β* and pro-IL-18 into IL-1*β* and IL-18 [50]. These inflammasome-activated cytokines play central roles in the propagation of the acute inflammatory response. IL-18 and caspase-1 play critical roles in the development of lung injury, and higher levels of IL-18 are correlated with disease severity and mortality in patients with ARDS [168]. Among 38 patients with acute respiratory failure, those with ARDS had significantly higher serum levels of IL-18, and serum IL-18 was significantly higher in nonsurvivors [51]. 

*(c) IL-6*. IL-6 is produced by a wide range of cells including monocytes/macrophages, endothelial cells, fibroblasts, and smooth muscle cells in response to stimulation by endotoxin, IL-1*β*, and TNF-*α* [52]. IL-6 is one of the most important mediators of fever and is critical for B-cell differentiation and maturation with secretion of immunoglobulins, cytotoxic T cell differentiation, macrophage and monocyte function, and production of acute phase proteins. Although IL-6 activates both proinflammatory and anti-inflammatory mechanisms, IL-6 primarily correlates with a proinflammatory profile during the early phase of ARDS. Plasma IL-6 increases early in patients at risk of developing ARDS [53]. IL-6 is elevated in both plasma and BAL fluid during ARDS [59] and correlates with mortality [54, 55]. 

*(d) IL-8*. IL-8 is a proinflammatory cytokine with a role in neutrophil/monocyte chemotaxis and neutrophil apoptosis inhibition. High plasma and BAL levels of IL-8 are found early during ARDS and predict outcome [169]. However, previous studies did not support such findings [170, 171]. In a recent monocenter study of 100 patients, only baseline IL-8 (among 6 other biomarkers) was associated with the development of multiorgan failure, even after adjustment for other relevant variables [32]. Also, several studies have evaluated the role of the anti-IL-8 autoantibody/IL-8 immune complexes in ARDS, a pathway that could lead to the identification of novel biomarkers and therapeutic targets [17, 172–175].

In a recent study, Calfee et al. used latent class analysis to integrate both clinical and biological data to identify two ARDS endotypes in an analysis of 1,022 patients from two ARDSNet trials (ARMA and ALVEOLI) [13]. A first endotype was categorized by more severe inflammation, as assessed by both IL-6 and IL-8 levels, and worse clinical outcomes, whereas a second endotype had less inflammation, less shock, and better clinical outcomes. Based on the data from the ALVEOLI trial, a “proinflammatory” endotype was associated with higher mortality and better response to higher levels of positive end-expiratory pressure [176].

*(2) Anti-Inflammatory Cytokines*. The inflammatory response is also strongly influenced by anti-inflammatory systems, including nonspecific (e.g., 2-macroglobulin, IL-10) and specific (e.g., IL-1 receptor antagonist (IL-1RA) antagonists, soluble IL-1 receptor II (sIL-1RII), soluble TNF receptor I (sTNF-RI), and soluble TNF receptor II (sTNF-RII)) of proinflammatory cytokines. 

*(a) IL-1RA*. Circulating IL-1RA levels are increased but do not predict the development of ARDS in at-risk patients [56]. Studies of gene expression in alveolar macrophages and circulating leukocytes from healthy control subjects and patients with ARDS revealed that sIL-1RII may be valuable as a biomarker because of increased levels in both the lung and circulation during ARDS [57]. 

*(b) sTNF-RI/sTNF-RII*. Soluble TNF-*α* receptors (sTNF-R) I and II can bind TNF and compete with its binding to the cellular receptor, thus reducing its bioavailability. Soluble TNF-RI and TNF-II are associated with morbidity and mortality in patients with ARDS [54], and a strategy of low tidal volume ventilation is associated with decreased sTNF-RI levels [54]. Trauma-associated ARDS differs clinically and biologically from ARDS due to other clinical disorders, with lower levels of sTNF-RI patients with trauma as a primary cause of ARDS [6, 58].

*(c) IL-10*. Interleukin-10 (IL-10) is an anti-inflammatory cytokine that is produced by several cells including B lymphocytes, monocytes, and alveolar macrophages [60]. Aside from inhibiting the production of IL-1 and TNF-*α*, IL-10 upregulates TNF receptors [177] and stimulates the production of the naturally occurring IL-1RA and the release of sTNF receptors [178]. IL-10 inhibits the production of proinflammatory mediators by alveolar macrophages involved during ARDS [179]. ARDS patients have lower plasma and BAL levels of IL-10 than at-risk patients who did not develop the syndrome [61]. Higher baseline IL-10 levels were associated with higher morbidity and mortality [59].

*(3) Additional Markers*. Other markers with potential clinical importance in ARDS-associated inflammation have been identified as putative biomarkers during ARDS.

High mobility group box nuclear protein 1 (HMGB1) is a DNA nuclear binding protein that is secreted by immune cells including monocytes and macrophages. HMGB1 increases early after severe trauma and correlates with systemic inflammatory response and development of ARDS [62]. Alveolar and plasma levels of HMGB1 (as measured in the arterial or central venous blood) are elevated in patients with ARDS and associated with outcome [109]. In 20 patients with ARDS, plasma levels of HMGB1 were also higher in nonsurvivors and correlated with levels of sRAGE [63].

Lipopolysaccharide binding protein (LBP) is an acute phase protein that is correlated with lung inflammation during ARDS [64]. More recently, it has also been demonstrated that serum levels of LBP were strongly associated with increased mortality and the development of ARDS in patients with severe sepsis [65].

Nitric oxide (NO) is a marker of oxidative stress that has also been investigated as a marker of ARDS. In patients with persistent ARDS, higher levels of nitric oxide and of its end-products (e.g., nitrotyrosine) are associated with mortality [66]. In contrast, higher urine NO levels were strongly associated with better clinical outcomes including mortality and ventilator-free days in patients enrolled in the ARDSNet low tidal volume trial [46]. Extracellular citrulline, the effective precursor of NO, is lower in the plasma from patients with severe sepsis and lower plasma citrulline is associated with the presence of ARDS [67]. Mechanisms involved in the regulation of lung injury by NO-dependent pathways remain unknown.

Although C-reactive protein (CRP) is widely considered as a marker of systemic inflammation, higher levels of CRP are associated with better outcome among patients with ARDS [68]. Nevertheless, a recent study found that albumin levels, rather than CRP, may help to predict and monitor the severity and course of ARDS in febrile critically ill patients with ARDS or at risk for the syndrome [69]. In the same study, levels of lactate dehydrogenase (LDH) predicted 28-day mortality but were not correlated with severity [69].

#### 3.1.3. Coagulation and Fibrinolysis

During early ARDS, activation of the inflammatory cascades results in the activation of the coagulation system, which in turn can influence inflammatory responses by affecting the expression of various cytokines such as IL-1, IL-6, and IL-8. Activation of coagulation pathways induces migration of inflammatory cells into alveoli through the endothelial and epithelial barriers and generates thrombin formation. In addition, proinflammatory events may also inhibit fibrinolysis and induce platelet activation [17].

Extravascular fibrin deposition, when localized predominantly in the alveolar compartment, is found in several acute inflammatory lung diseases, and enhanced alveolar procoagulant activity is reported in ARDS patients [180]. Fibrin deposition may be beneficial for gas exchange by sealing leakage sites when lung capillary endothelial and epithelial barriers are disrupted. Nevertheless, fibrin alveolar deposition may be harmful since it can lead to activation of neutrophils and fibroblasts, endothelial injury, loss of surfactant activity favoring alveolar collapse, impaired alveolar fluid clearance, and thrombotic obstruction of the microcirculation [180].

*(1) PAI-1*. The balance between activation of coagulation and activation of fibrinolysis is an important determinant of the amount and duration of fibrin deposition during lung injury. Plasminogen activator (PA) and plasminogen activator inhibitor-1 (PAI-1) regulate fibrinolysis through the conversion of plasminogen to plasmin, a fibrinolytic enzyme. PA-1 is a major endogenous inhibitor of PA. Both PA and PA-1 are secreted by various cells including macrophages, fibroblasts, and lung endothelial and epithelial cells [71]. During ARDS, alveolar epithelial cells and activated macrophages overexpress PAI-1, thus contributing to decreased alveolar fibrinolytic activity. Nevertheless, the value of PAI-1 as a biomarker in ARDS remains controversial. PAI-1 levels were higher in patients with ARDS than in patients with hydrostatic lung edema [72], and higher PAI-1 levels are associated with mortality and higher durations of mechanical ventilation in patients with ARDS [72, 169]. In a large Finnish study, PAI-1 levels were not correlated with mortality or development of ARDS in critically ill patients under mechanical ventilation, but low baseline plasma levels of the soluble urokinase plasminogen activator receptor (suPAR) were predictive of survival [181]. In a secondary analysis of two large randomized controlled trials, PAI-1 was associated with lung injury (as defined as decreased oxygenation index) but not with mortality [28]. Nevertheless, in another study of patients from the ARDSNet ARMA study, higher levels of PAI-1 were independently associated with higher mortality and clinical outcomes, including organ failure [182]. However, this association between PAI-1 levels and the development of multiorgan failure was not confirmed in a recent study of 100 ARDS patients [32].

*(2) Protein C*. Protein C system is an important endogenous regulator of coagulation and fibrinolysis. Protein C is synthesized by the liver and circulates as an inactive compound. It is transformed to its active form on cell surface by the thrombomodulin- (TM-) thrombin complex [75, 76]. The endothelial cell protein C receptor (EPCR) is another cell surface protein that can further enhance protein activation by binding the TM-thrombin complex [183]. In addition to suppressing thrombin formation, activated protein C has anti-inflammatory properties such as decreasing the levels of proinflammatory cytokines [184]. Protein C can improve endothelial permeability and exert antiapoptotic effects via p53 pathways [185]. Activated protein C can also inactivate PAI-1, thus promoting fibrinolysis [186]. Plasma protein C was significantly lower in patients with ARDS as compared to controls, and it was associated with worse clinical outcomes, including higher hospital mortality, shorter duration of unassisted ventilation, and increased risk of multiple organ failure [73]. In a larger cohort of patients with early ARDS, low plasma levels of protein C were again associated with mortality and adverse clinical outcomes [182]. Decreased levels of protein C during ARDS suggest a link between hypercoagulability and mortality.

*(3) Thrombomodulin*. Thrombomodulin (TM) is a multidomain transmembrane-bound glycoprotein found on the surface of endothelial cell. Its main role is to neutralize the procoagulant effects of thrombin and accelerate activation of protein C. In addition to its membrane-bound form, TM also exists as a circulating soluble isoform in the plasma. In patients with ARDS, levels of soluble thrombomodulin (sTM) are higher in the pulmonary edema fluid than in plasma [73], suggesting an alveolar source, but no correlation was found between plasma sTM and the development of ARDS, yet higher levels of sTM were observed in patients at high risk for ARDS. In patients with established ARDS, higher plasma and alveolar levels of sTM were correlated with severity of illness and multiple organ failure [73]. In a larger analysis of 449 patients, elevated levels of plasma sTM were associated with increased mortality thus possibly reflecting an increased degree of inflammation and both lung and systemic endothelial damage [74].

*(4) Tissue Factor (TF)*. Tissue factor (TF) is a 47 kDa transmembrane glycoprotein that is the most potent stimulator of the extrinsic coagulation cascade. TF initiates the coagulation cascade by binding and allosterically activating coagulation factor VIIa. The resulting TF-VIIa complex binds the substrate coagulation factor X via multiple interactions along an extended interface to produce the TF-VIIa-X complex. This complex leads eventually to thrombin formation and fibrin deposition [77]. Levels of TF in lung edema fluid are higher than plasma levels in patients with ARDS, supporting a lung origin for TF in this setting, and both plasma and alveolar levels of TF are higher in ARDS patients as compared to patients with hydrostatic edema [187]. Notably, patients with sepsis-induced ARDS may have higher levels of TF as compared to patients without ARDS [78]. 

*(5) Cell-Free Hb*. Levels of cell-free hemoglobin (Hb) are higher in the air space of ARDS patients as compared to critically ill patients with hydrostatic lung edema [79]. Instillation of red blood cells or cell-free hemoglobin causes lung injury in rats [188] and intra-alveolar hemorrhage is associated with high levels of intra-alveolar cell-free Hb, more severe lung injury, and increased lipid peroxidation in the lung from mice with tissue factor deficiency [79]. Precise cellular and molecular mechanisms by which cell-free hemoglobin in the air space could mediate or potentiate ARDS are currently under investigation [189]. Cell-free Hb could activate chemokine release within the lung, and decompartmentalization of hemoglobin is likely to provide a significant proinflammatory stimulus in the setting of diffuse alveolar damage and hemorrhage during ARDS [80].

#### 3.1.4. EF/PL Protein Ratio

The pathophysiology of ARDS includes disruption of several physical barriers including endothelial and epithelial cell layers, the basement membrane, and the extracellular matrix, resulting in increased pulmonary microvascular permeability. The pulmonary edema fluid-to-plasma protein (EF/PL) ratio is a rapid, safe, and noninvasive measure of alveolar-capillary membrane permeability. The EF/PL ratio was first proposed as a tool to determine the etiology of acute pulmonary edema [81]. More recently, in a large study of 390 critically ill patients, Ware et al. demonstrated that the EF/PL ratio had an excellent discriminative value in distinguishing ARDS from hydrostatic edema and was strongly associated with clinical outcomes. Using a cutoff of 0.65, the EF/PL ratio had a sensitivity of 81% and a specificity of 81% for the diagnosis of ARDS [82].

### 3.2. Fibroproliferative Phase of ARDS

In some patients, important and persistent accumulation of macrophages, fibrocytes, fibroblasts, and myofibroblasts in the alveolar compartment leads to excessive deposition of ECM components including fibronectin and collagen types I and III, among other proteins. An imbalance between profibrotic and antifibrotic mediators may subsequently drive this fibroproliferative response [190]. Growth factors play a major role in the resolution of ARDS [17]. Lung endothelial repair is promoted by vascular endothelial growth factor (VEGF). A variety of growth factors promote repair of the alveolar epithelium including keratinocyte growth factor (KGF), hepatocyte growth factor (HGF), fibroblast growth factor (FGF), and transforming growth factor-*α* (TGF-*α*) [17]. Two major pathways with opposite effects involve growth factors during ARDS: tyrosine kinase receptor mediation (e.g., KGF, HGF, FGF, and VEGF) and serine-threonine kinase receptors such as TGF-*β*1, which tend to have opposed effect on the upregulation that occurs when the tyrosine kinase receptor pathway is involved [85, 191, 192].

#### 3.2.1. Endothelial Proliferation

Novel evidence points to a potential role of VEGF in promoting repair of the alveolar-capillary membrane during recovery from ARDS, and understanding the role of VEGF in this disease process could be crucial for developing new therapeutic strategies [85, 193]. In the lung, VEGF is produced primarily by epithelial cells; it increases microvascular permeability [83] but has an important role also during the repair phase by stimulating endothelial cell proliferation and survival [86, 149]. The levels of VEGF are increased in plasma from patients with ARDS but are decreased in BAL fluid, compared to healthy controls; subsequently, BAL levels of VEGF increase during the resolution of lung injury [39, 84, 149].

#### 3.2.2. Epithelial Proliferation and Apoptosis

KGF, also known as FGF-7, is a potent mitogenic factor for alveolar epithelial cells that is primarily produced by fibroblasts and other cells such as T lymphocytes. KGF regulates transepithelial transport of sodium by stimulating the epithelial channel Na^+^-K^+^-ATPase in alveolar epithelial cells [87].

HGF is a nonspecific mitogen secreted by fibroblasts, alveolar macrophages, endothelial cells, and epithelial cells. Several animal and human studies suggest that KGF and HGF could protect the alveolar space against injury and could facilitate the repair of alveolar structures after injury [88, 194, 195]. KGF levels could be measured in the BAL from patients with ARDS but not in the BAL from those without ARDS; in addition, BAL KGF was associated with poor prognosis [196]. Elevated HGF levels were also associated with outcome [196]. In 36 patients with ARDS and 11 patients with hydrostatic lung edema, HGF and KGF were proven biologically active in the edema fluid of patients with ARDS, and higher levels of HGF were associated with mortality in these patients [197].

Apoptosis of alveolar epithelial cells is a major phenomenon in the initiation and perpetuation of lung injury [89]. The Fas/FasL system plays an important role in the regulation of cell life and death through its ability to initiate apoptosis [198]. This system combines the cell membrane surface receptor Fas (CD95) and its natural ligand FasL (CD95L). Membrane-bound FasL mediates lymphocyte-dependent cytotoxicity, clonal deletion of alloreactive T cells, and activation-induced suicide of T cells [199]. Its soluble form (sFasL) results from cleavage of membrane FasL by MMPs and induces apoptosis in susceptible cells [200]. Apoptosis is induced when membrane-bound or soluble FasL binds to Fas-bearing cells. By contrast, apoptosis is inhibited when soluble Fas binds to either membrane-bound FasL or sFasL thus preventing FasL from interacting with membrane-bound Fas receptors [201]. In patients with ARDS, sFasL was detectable in the lung before and after the onset of clinically defined ARDS, and nonsurvivors had significantly higher BAL levels of sFasL on day 1 as compared with survivors [200]. Both soluble Fas and soluble FasL were associated with outcome and higher in the lung edema fluid from patients with ARDS, compared to control patients with hydrostatic pulmonary edema [202]. Nevertheless, recent findings suggested limited role for Fas/FasL system and apoptosis in airway epithelial cell death during ARDS [90].

#### 3.2.3. Fibroblast Proliferation

Pulmonary fibroblasts produce procollagen III peptide (PCP-III), that is, a precursor of collagen. The NT part of procollagen III, resulting from the enzymatic cleavage of procollagen by specific proteases in the extracellular space, is considered as a marker of collagen synthesis. Alveolar levels of N-PCP-III are higher in ARDS patient, as compared with controls [203]. The elevation of N-PCP-III in pulmonary edema fluid begins within the first 24 h of ARDS, that is, during the acute phase of increased endothelial and epithelial permeability to protein, suggesting that fibrosing alveolitis could begin very early in the course of clinical ARDS [203]. In another study, high levels of N-PCP-III were early predictors of poor outcome [204, 205]. More recently, Forel et al. measured alveolar N-PCP-III in patients with nonresolving ARDS, thus identifying patients who had developed lung fibroproliferation [206]. Unfortunately, it is still unknown whether N-PCP-III measurements could be useful in selecting patients who would benefit from glucocorticoid therapy, among others, to reduce the ARDS-associated lung fibrosis [207].

## 4. Perspectives

### 4.1. Combining Biomarkers

Despite advances in the identification of biomarker candidate and better understanding of ARDS pathogenesis, no single clinical or biological marker reliably predicts clinical outcomes in ARDS. The combination of clinical and biological marker is attractive in order to improve the sensitivity and/or the specificity of the test, especially through a recent approach aimed at measuring 8 biological markers that reflect endothelial and epithelial injury, inflammation, and coagulation: vWF, SP-D, TNF-R1, IL-6, IL-8, ICAM-1, protein C, and PAI-1 in 549 patients enrolled in the the ARDSNet trial of low versus high positive end-expiratory pressure [208]. Clinical predictors predicted mortality with an area under the ROC curve (AUC) of 0.82, whereas a combination of these 8 biomarkers and the clinical predictors had an AUC of 0.85. The best performing biomarkers were the neutrophil chemotactic factor IL-8 and SP-D, a product of AT2 cells [208], supporting the concept that acute inflammation and alveolar epithelial injury are important pathogenetic pathways in human ARDS. More recently, a panel of biomarkers of lung epithelial injury and inflammation (SP-D, sRAGE, IL-8, CC16, and IL-6) provided excellent discrimination for diagnosis of ARDS in patients with severe sepsis [20]. Therefore, and beyond their better diagnostic and prognostic values, the use of such biomarker panels may be useful for selecting patients for clinical trials that are designed to reduce lung epithelial injury [113]. Nevertheless, whether a therapeutic strategy based on biomarker measurements would benefit patient outcome has never been investigated.

### 4.2. Lung Imaging as an ARDS Biomarker

Studies of lung imaging during ARDS have revealed that adequate ventilator settings may vary among patients with the same syndrome. In a large study, gas and tissue distribution in the lungs of ARDS patients were assessed using computed tomography (CT) and compared to those of healthy volunteers [11]. Lung morphology in ARDS is characterized by marked excess of lung tissue associated with a major decrease in aerated lung regions and in functional residual capacity. Some patients with ARDS exhibit preserved aeration of the upper lobes despite the presence of an overall excess of lung tissue (“focal” ARDS), as opposed to other patients with more diffuse loss of aeration and excessive lung tissue (“diffuse” or “nonfocal” ARDS) [209].

Lung morphology may influence the response to positive end-expiratory pressure (PEEP), recruitment maneuvers (RM), prone position, and patient outcome [12, 210]. In a prospective study of nineteen patients with ARDS, Constantin et al. found that lung morphology at zero end-expiratory pressure could predict the response to a RM with continuous positive airway pressure of 40 cm H_2_O for 40 seconds. Nonfocal morphology was associated with higher lung recruitability and PaO_2_/FiO_2_ was significantly increased by the RM [12, 211]. In contrast, patients with focal lung morphology were at risk of significant hyperinflation during the RM, with no improvement of arterial oxygenation.

It has also been hypothesized that the effects of PEEP may depend on lung morphology. Puybasset et al. assessed the responses to PEEP among patients with focal or nonfocal ARDS [210]. The regional distribution of intrapulmonary gas and lung tissue influences the effects of PEEP in ARDS patients: maximal alveolar recruitment, without evidence of overdistension, was observed in patients with nonfocal ARDS. Nevertheless, PEEP induced mild alveolar recruitment in patients with focal ARDS, along with overdistension of previously aerated lung regions.

Interestingly, phenotyping patients with ARDS based on their lung morphology might be possible by measuring plasma sRAGE with commercially available kits, even though these findings need further validation [100, 112]. However, RAGE pathway is a promising candidate for subphenotyping patients with ARDS, as it is believed to play a major role in the mechanisms leading to AFC and their regulation [110]. Recent findings that support a relationship between impaired AFC and lung morphology may therefore fill a gap in the full recognition of an ARDS phenotype based on lung morphology that could be linked to an endotype of impaired AFC and activated RAGE pathway [12, 100, 101, 112, 113, 212].

### 4.3. Biomarkers in ARDS: Can They Improve Patient Care?

Biomarkers are broadly used in critically ill patients, especially during inflammatory and/or infectious diseases. Biomarkers have been commonly defined as characteristics that are objectively measured and evaluated as indicators of normal biological processes, pathogenic processes, or pharmacologic responses to therapeutic interventions [213, 214]. Biomarkers provide a powerful approach to understand a disease with multiple applications in observational and analytic epidemiology, randomized clinical trials, screening, and diagnosis or prognosis [215].

Nevertheless, there are important technical attributes for a relevant biomarker. First, the marker must be present in peripheral body tissue and/or fluid (e.g., blood, urine, saliva, breath, or cerebrospinal fluid); second, it must be easy to detect or quantify in assays that are both affordable and robust; and, third, its regulation should be associated as specifically as possible with damage of a particular tissue, preferably in a quantifiable manner. Prior to the widespread use of a marker of interest, it is essential that validation and confirmation of candidate biomarkers by robust statistical methods are performed during biomarker discovery [215]. Sensitivity and specificity are common quality parameters for biomarkers. Sensitivity describes the probability of a positive test in cases and specificity describes probability of negative test in controls. An association between sensitivity and specificity is represented in the ROC curve by graphing sensitivity versus 100 − specificity. Area under the ROC curve (AUROC) is therefore a measure of performance of a marker. There is no absolute cutoff value of AUROC for robustness of a marker, but a minimum of 0.7 is required and values greater than 0.8 are good particularly in a heterogeneous critically ill patient population [216, 217].

To summarize, an ideal biomarker should indicate a clear relationship with the pathophysiologic event, needs to be reliable, reproducible, disease specific, and sensitive, and should be sampled by simple methods and relatively inexpensive, with little or no diurnal variation. During ARDS, no single marker has been validated with all these criteria to date, yet we believe that sRAGE may fulfill all prerequisites of a biomarker of ARDS. First, plasma sRAGE has good diagnostic and prognostic values [96, 99, 100, 109, 112]. Second, it is very well correlated with lung injury severity and specific pathophysiologic features of ARDS, for example, alveolar fluid clearance and lung morphology [96, 99–101, 109, 112]. Previous studies of the predictive value of early levels of sRAGE for the development of ARDS in a general population of patients admitted to the emergency department were negative [130], but current research focuses on both the kinetics of sRAGE and esRAGE and RAGE gene polymorphisms as predictors of the development of ARDS in at-risk critically ill patients [114]. Finally, there is some evidence suggesting that monitoring sRAGE could inform, at least partially, on therapeutic responses in patients with or without ARDS [24, 99, 113]. If these data are confirmed by future studies, such findings would definitely help to reinforce sRAGE a real biomarker of ARDS.

## 5. Conclusion

Biomarker research provides an important translational link to our understanding of lung pathobiology. Through the identification and testing of candidate biomarkers, we have gained insight into the pathogenic importance of endothelial and epithelial injury and have started to unravel the complex pathways that contribute to endothelial and epithelial cell dysfunction, inflammation, fibrosis, and apoptosis in ARDS. In addition, biomarker studies may help us to explore the cellular and molecular mechanisms of various therapeutic strategies for ARDS, and to better understand the potential proinflammatory effects of mechanical ventilation. Given the clinical heterogeneity of patients with ARDS and the complexity of the underlying pathobiology, it is unlikely that a single biomarker will emerge for ARDS, as cardiac-specific troponin did for myocardial infarction, but the development of small biomarker panels reflecting each important lung injury pathway would provide valuable predictive and prognostic information for both clinicians and investigators. While biomarkers are currently not recommended for use in clinical practice in ARDS, biomarker discovery may hold significant promise in order to develop and apply targeted therapies, and to identify candidates for enrollment in patient-tailored clinical trials of novel therapies for ARDS.

## Figures and Tables

**Table 1:** Biomarkers of acute respiratory distress syndrome organized by phases and pathways of lung injury.

Pathophysiologic feature of ARDS	Biomarker	References
Exudative phase		
*Epithelium damage*		
(i) Alveolar type 1 cells	RAGE	[22–24]
	HTI_56_	[25, 26]
(ii) Alveolar type 2 cells	Surfactant	[27, 28]
	KL-6	[29, 30]
(iii) Clara cells	CC16	[31, 32]
*Endothelium damage*		
	Ang-1, Ang-2	[33, 34]
	ICAM-1	[35, 138–140]
	Selectins	[36, 37]
	VEGF	[38, 39]
	vWF	[40–42]
*Lung matrix alteration*		
	Laminin	[43, 44]
	Elastin/desmosine	[45, 46]
	MMPs	[47–49]
*Inflammatory cascade*		
(i) Proinflammatory	IL-1*β*/TNF-*α*	[17, 161–164, 167]
	IL-18	[50, 51]
	IL-6	[52–55]
	IL-8	[169–171]
(ii) Anti-inflammatory	IL-1RA	[56, 57]
	sTNF-RI/sTNF-RII	[6, 54, 58]
	IL-10	[59–61]
(iii) Additional markers	HMGB1	[62, 63]
	LBP	[64, 65]
	NO	[46, 66, 67]
	CRP	[68]
	Albumin	[69]
	LDH	[69]
*Coagulation and fibrinolysis*		
	PA-1	[32, 53, 70–72]
	Protein C	[75, 76, 182]
	Thrombomodulin	[73, 74]
	Tissue factor	[75–78]
	Cell-free hemoglobin	[75, 76, 79, 80]
*Pulmonary vascular permeability*		
	EF/PL ratio	[81, 82]

Fibroproliferative phase		
*Endothelial proliferation*		
	VEGF	[39, 83–86]
*Epithelial proliferation*		
	KGF	[87]
	HGF	[88, 194, 195, 197]
*Epithelial apoptosis*		
	Fas/FasL	[25, 88–91]
*Fibroblast proliferation*		
	N-PCP-III	[88, 194, 195, 203, 207]
